# Protopanaxadiol and Protopanaxatriol-Type Saponins Ameliorate Glucose and Lipid Metabolism in Type 2 Diabetes Mellitus in High-Fat Diet/Streptozocin-Induced Mice

**DOI:** 10.3389/fphar.2017.00506

**Published:** 2017-08-02

**Authors:** Jianjun Deng, Yao Liu, Zhiguang Duan, Chenhui Zhu, Junfeng Hui, Yu Mi, Pei Ma, Xiaoxuan Ma, Daidi Fan, Haixia Yang

**Affiliations:** ^1^Shaanxi Key Laboratory of Degradable Biomedical Materials, School of Chemical Engineering, Department of Food Science and Engineering, Northwest University Shaanxi, China; ^2^Shaanxi R&D Center of Biomaterials and Fermentation Engineering, School of Chemical Engineering, Northwest University Shaanxi, China; ^3^Department of Nutrition and Food Safety, College of Public Health, Xi’an Jiaotong University Shaanxi, China

**Keywords:** protopanaxadiol-type saponins, protopanaxatriol-type saponins, type 2 diabetes mellitus, inflammation, glucose metabolism, lipid metabolism

## Abstract

Ginsenoside is a major active component of ginseng, which exhibits various pharmacological properties such as hepatoprotection, tumor suppression and diabetes resistance. In this study, the anti-diabetic effects of protopanaxadiol (PPD) and protopanaxatriol (PPT)-type saponins were explored and compared in high-fat diet/streptozocin-induced type 2 diabetes mellitus (T2DM) mice. Our results showed that low or high dose (50 mg/kg bodyweight or 150 mg/kg bodyweight) PPD and PPT significantly reduced fasting blood glucose, improved glucose tolerance and insulin resistance in T2DM mice. PPD and PPT also regulated serum lipid-related markers such as reduced total cholesterol (TC), triglyceride (TG), and low-density lipoprotein cholesterol in T2DM mice. In addition, PPD and PPT dramatically ameliorated the inflammatory responses by suppressing the secretion of pro-inflammatory cytokines like tumor necrosis factor-alpha and interleukin-6 in serum level and gene expression in liver level, and improved the antioxidant capacity by increasing the superoxide dismutase and decreasing malondialdehyde levels in the serum of T2DM mice. Moreover, the anti-diabetic effect of PPD and PPT appeared to be partially mediated by the suppression of hepatic metabolism genes expression such as peroxisome proliferator-activated receptor gamma coactivator 1-alpha, phosphoenolpyruvate carboxykinase, and glucose-6-phosphatase, as well as facilitating lipid metabolism genes expression such as microsomal TG transfer protein in the liver tissues of T2DM mice. Taken together, our results indicated that PPD and PPT might potentially act as natural anti-diabetic compounds to be used for preventing and treating the T2DM and its complications in the future.

## Introduction

Type 2 diabetes mellitus is a chronic metabolic disease that can impose serious damage on human health and the quality of life (Whiting et al., 2011; Ji et al., 2015). Recently, a survey estimated that there will be more than 642 million people suffering from diabetes in nearly all countries by the year 2040 (Cho, 2016). In a cyclical manner, hyperglycemia also increases diabetes complications by further aggravating the condition, such as hypoglycemia, ketoacidosis, neuropathy, nephropathy, cardiopathy and retinopathy (Niu et al., 2012). Currently, the majority of oral hypoglycemic drugs used for the treatment of diabetes have demonstrated side effects and adverse reactions (Li et al., 2016). Therefore, one of the major research areas is discovering and developing alternate drugs with fewer side effects. Medicinal herbs were applied to treat wide range of diseases including diabetic mellitus for a long time before the birth of Western Medicine (Basch et al., 2003), and it was well known that their therapeutic function usually accompanied with less side effects compared with the chemical agents such as phenformin and tolbutamide, indicating the use of traditional herbal medicine, which provoked the interest of many researchers worldwide (Yuan et al., 2012).

Ginseng has been used as a herbal medicine in China for thousands of years and proved to exhibit wide pharmacological properties, such as anti-cancer, anti-diabetes, anti-fatigue, anti-aging, hepatoprotective, and neuroprotective effects (Li et al., 2012a,b). The clinical trials also indicated that ginsenoside possessed antihyperglycemic activity and played an important role in the management of diabetic mellitus and its complications (Sotaniemi et al., 1995; Ma et al., 2008; Shishtar et al., 2014). Ginsenoside monomers, such as ginsenoside Rg1, Re, Rb1, Rk1, Rg5, Rg3, Rb2, and Rh2, have wonderful hypoglycemic effect in the diabetic mice models (Cho et al., 2006a; Park et al., 2008; Wang et al., 2009; Lee et al., 2011; Whiting et al., 2011; Li et al., 2016; Teng et al., 2017). However, it is difficult to obtain higher quality ginsenoside monomers in terms of purity and solubility, and increased quantity, for patient administration. Therefore, it is also necessary to study the anti-diabetic activity of the ginsenoside mixture, especially protopanaxadiol (PPD) and protopanaxatriol (PPT)-type saponins, which are two important types of the ginsenoside. According to the chemical structural difference of aglycones, ginsenoside can be classified into two main categories: PPD (e.g., Rb1, Rb2, Rb3, Rc, Rd, Rg5, Rk1, and Rg3) and PPT (e.g., Re, Rf, Rk3, Rh4, Rh1, and Rg1) classifications (Qi et al., 2011). However, little is known about the anti-diabetic effect of PPD and PPT and its potential underlying mechanisms. Here, the aim of the present study was to explore and compare the effects of PPD and PPT on glucose and lipid metabolism in high-fat diet/streptozocin (STZ)-induced T2DM mice and their underlying mechanisms of action.

## Materials and Methods

### HPLC Analysis of Ginsenoside PPD and PPT

Standard ginsenoside including Rb1, Re, Rg3, Rg5, Rk1, Rk3, Rh1, and Rh4 were purchased from Purification Technology Development Co., Ltd (Chengdu, China). PPD and PPT were synthesized from Rb1 and Re, respectively, and assayed by HPLC (SSI, United States) on a Poroshell 120 EC-C18 column (5 μm, 250 mm × 4.6 mm, OmniGene LLC) at 203 nm. The gradient elution solvent consisted of water (A) and acetonitrile (B). The process of elution was carried out as follows: 0–20 min, 80% A, 20% B; 20–45 min, 80–54% A, 20–46% B; 45–55 min, 54–45% A, 46–55% B; 55–80 min, 45% A, 55% B. The column was eluted at 25°C with a linear gradient of acetonitrile/water from 80:20 to 45:55 (v/v) at a flow rate of 1.5 mL/min, and then the extract were subsequently filtered and lyophilized. The stock solution of PPD/PPT was prepared containing 8% emulsifier EL (Jiangxi Yipusheng Pharmaceutical Co., Ltd, Jiangxi, China), 10% ethyl alcohol (Tianli Chemical Reagent Co., Ltd, Tianjin, China), and 82% saline (Xi’an Jingxi Shuanghe Pharmaceutical Co., Ltd., Shaanxi, China) for the intraperitoneal injection.

### Animals and Diets

**FIGURE 1:**
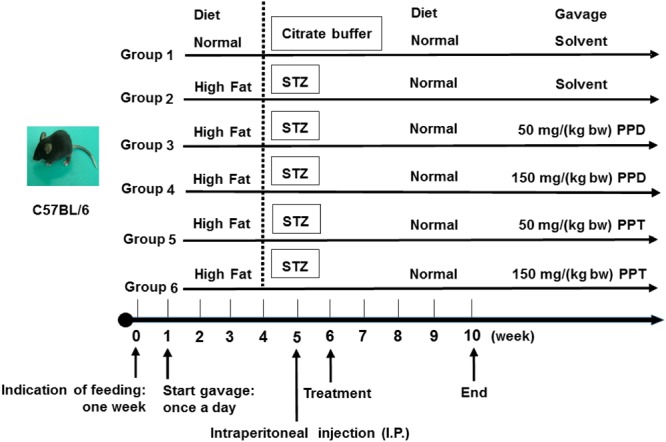
Animal experimental design. Group 1, NDC group; Group 2, DC group; Group 3, LPPD group; Group 4, HPPD group; Group 5, LPPT group; Group 6, HPPT group.

C57BL/6 mice (6-week-old, 20 ± 2 g male) were supplied by the Experimental Animal Center of the Fourth Military Medical University and housed in a controlled room (constant temperature 25 ± 2°C and humidity 55 ± 5%, 12 h light/dark cycle). The animal experimental design is summarized in **Figure 1**. All experiments were performed in compliance with the relevant laws and institutional guidelines, and conducted with the approval of the Northwest University Animal Ethics Committee.

Mice were given the first week to acclimate to their new environment and were given a normal diet. Following the acclimation period, 60 mice were randomly divided into six groups (*n* = 10). Group 1 was given a normal diet (Chow diet, 10% of calories derived from fat, 3.85 kcal/gm, Research Diets, D12450B) with adlibitum access to food and water, and designated as the control group (NDC). Group 2 was given a diabetic diet (60% of calories derived from fat, 5.24 kcal/gm, Research Diets, New Brunswick, NJ, United States, D12492) for 4 weeks, and designated as the diabetic control (DC). Group 3 and Group 4 were given the diabetic diet for 4 weeks and treated with 50 and 150 mg/kg bodyweight of PPD, and designated as the LPPD and HPPD, respectively. Group 5 and Group 6 were given the diabetic diet for 4 weeks and treated with 50 and 150 mg/kg bodyweight of PPT, and designated as the LPPT and HPPT, respectively. The diet, behavior, and appearance of the all mice were recorded daily, and the body weight, food intake, and FBG levels were investigated weekly. On the first day of week 5, mice were administered a dose of 30 mg/kg bodyweight of STZ (Sigma, St Louis, MO, United States) which dissolved in 100 mM citrate buffer via intraperitoneal injection for 4 days. The NDC group was treated with citrate buffer. Exception of the NDC group, FBG levels in other groups more than 11.0 mM after one week STZ injection were confirmed hyperglycemic. From 6 to 10 weeks, mice in groups 3 through 6 were treated with the indicated dose of ginsenoside once a day.

### Fasting Blood Glucose, Oral Glucose Tolerance, and Insulin Tolerance Test

The FBG was monitored weekly from the initiation of experiment, and measured using a glucometer (ACCU-CHEK, Roche, Germany). The OGTT was conducted in week 9. Following a 16 h fast, mice were administered an oral glucose solution of 1.8 g/kg bodyweight via a gastric tube. Blood samples were collected from the tail vein, and the glucose concentrations were measured at 0 (basal blood glucose levels), 30, 60, 90, and 120 min post-glucose administration. The ITT was given 5 days prior to the completion of the study period. Following a 6 h fast, mice were intraperitoneally injected with 0.8 U/kg bodyweight insulin. Blood samples were collected from the tail vein, and glucose levels were measured at 0, 15, 30, 60, 90, and 120 min post-injection. The AUC for OGTT and ITT was calculated at 0, 30, 60, 90, and 120 min post glucose/insulin administration using the trapezoidal method.

### Blood and Tissue Collection

At the completion of the 4 weeks treatment, the mice were fasted for 12 h prior to being sacrificed. Anesthesia was administered via intraperitoneal injection of 50 mg/kg bodyweight pentobarbital sodium. Whole blood samples were collected retro-orbitally, and the serum was isolated by centrifugation at 3500 *g* for 10 min and stored at –80°C. The liver and pancreas were removed, washed, and fixed in 10% formalin solution for histopathological analysis.

### Biochemical Parameters

Serum insulin levels were measured with rat insulin ELISA kit (Crystal Chem, Downers Grove, IL, United States). The levels of SOD, malondialdehyde (MDA), TC, and TG were determined by a commercial kit (Nanjing Jiancheng Biology Engineering Institute, Nanjing, China). The SOD activity was defined as the amount of enzymatic reaction in 1 mL of serum per min. HDL-C and LDL-C levels were measured using colorimetric enzyme kits (Sigma–Aldrich, MO, United States). The secretion levels of tumor necrosis factor alpha (TNF-α) and IL-6 were measured using ELISA kits (Abcam, Cambridge, United Kingdom). The HOMA-IR was calculated as HOMA-IR = FBG × fasting insulin level/22.5.

### Isolation of Total RNA and Real-Time PCR Assay

Total RNA was isolated from the liver using a Trizol reagent (TIANGEN, Life Technologies Corporation 92008, United States). Total RNA (1.0 μg) was reverse transcribed to cDNA using the Thermo Scientific Revert Aid First Strand cDNA Synthesis Kit (Thermo Fisher Scientific Inc., MA, United States). The mRNA expression was quantified by the Light Cycler 480 real-time system (Roche, Switzerland) using the SYBR green Master I kit (Roche, Switzerland). Relative expression level of target mRNA was normalized to glyceraldehyde 3-phosphate dehydrogenase (GAPDH) mRNA by the 2^-ΔΔCT^ method. The primer sequences used are listed in **Table 1**.

**Table 1:** The primer sequences were used for qPCR in this study.

	Forward (5’ to 3’)	Reverse (5’ to 3’)	Reference
PGC-1α	AAGTGTGGAACTCTCTGGAACTG	GGGTTATCTTGGTTGGCTTTATG	Zhang et al., 2016
PEPCK	TGCCTCTCTCCACACCATTGC	TGCCTTCCACGAACTTCCTCAC	Zhang et al., 2016
G6Pase	CGACTCGCTATCTCCAAGTGA	GTTGAACCAGTCTCCGACCA	Zhanget al., 2016
TNF-α	CCTGTAGCCCACGTCGTAG	GGGAGTAGACAAGGTACAACCC	Zhang et al., 2016
IL-6	CAGTTGCCTTCTTGGGACTGA	ACAGGTCTGTTGGGAGTGGT	Zhang et al., 2016
MTTP GAPDH	AAGCAGAAATTAGAGCTGAAGAC GGAGCGAGATCCCTCCAAAAT	CAGTGGCTCTGGAAGACCT GGCTGTTGTCATACTTCTCATGG	This work This work

### Histological Analysis

For H&E staining, liver and pancreas tissues were fixed in 10% formaldehyde and embedded in paraffin wax. The paraffin-embedded liver and pancreas samples were cut into 5 μm sections. The sections were stained with H&E, and subsequently examined using a light microscope for histopathological examination.

### Statistical Analysis

All data were expressed as the mean ± standard error of mean (SEM). The statistical analysis of the experimental results was analyzed using the SPSS 15.0 (SPSS Inc., Chicago, IL, United States) for windows statistical program. All graphical representations were performed with GraphPad Prism software (Version 5.0, GraphPad Software Inc., San Diego, CA, United States) using one-way analysis of variance (ANOVA) with Tukey’s *post hoc* analysis. A *p*-value of <0.05 was considered statistically significant. *p* < 0.05, *p* < 0.01, and *p* < 0.001 were highlighted with ^∗^, ^∗∗^, and ^∗∗∗^, respectively.

## Results

### HPLC Analysis and Identification of Ginsenoside PPD and PPT

**FIGURE 2:**
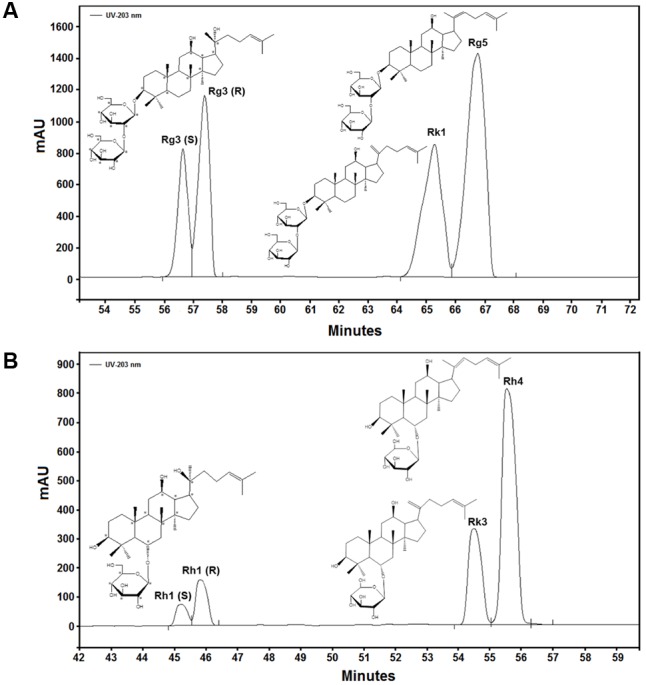
HPLC chromatograms of PPD **(A)** and PPT **(B)**.

To identify the main composition present in PPD and PPT, we performed an HPLC analysis (**Figures 2A,B**) and the corresponding contents have been summarized in **Table 2**. The different compounds were quantified by comparing the tR with respect to ginsenoside standards. The results indicated that there are four kinds of ginsenoside existing in PPD and PPT, respectively. The tR of each composition and corresponding contents were as follows: for PPD: 56.66 min, ginsenoside Rg3 (S); 57.40 min, ginsenoside Rg3 (R); 65.29 min, ginsenoside Rk1; and 66.77 min, ginsenoside Rg5. For PPT: 45.23 min, ginsenoside Rh1 (S); 45.80 min, ginsenoside Rh1 (R); 54.50 min, ginsenoside Rk3; and 56.32 min, ginsenoside Rh4.

**Table 2:** Composition and content of PPD and PPT analyzed by HPLC*^a^*.

Compounds	Retention time (tR) (min)	Content (mg/g)
Rg3 (S)	56.66	12.86 ± 0.43
Rg3 (R)	57.40	20.20 ± 0.59
Rk1	65.29	25.24 ± 0.75
Rg5	66.77	41.69 ± 0.92
Rh1 (S)	45.23	4.51 ± 0.47
Rh1 (R)	45.80	9.85 ± 1.49
Rk3	54.50	24.01 ± 4.96
Rh4	56.53	61.44 ± 0.74

*^a^Values are the means ± standard deviations (*n* = 3).*

### Effects of PPD and PPT on Body Weight and Food Intake

**FIGURE 3:**
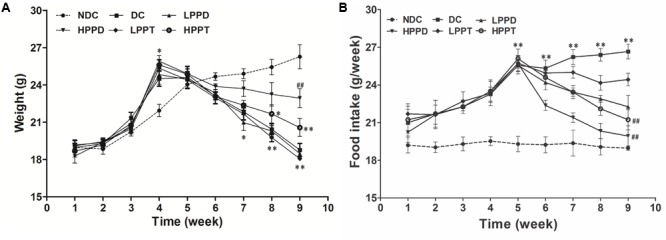
Effects of PPD and PPT on **(A)** body weight and **(B)** food intake in high-fat diet and STZ-induced diabetic mice during the whole feeding period. ^∗^*p* < 0.05, ^∗∗^*p* < 0.01, ^∗∗∗^*p* < 0.001 vs. NDC group; ^#^*p* < 0.05, ^##^*p* < 0.01, ^###^*p* < 0.001 vs. DC group. Different groups were significantly different (*p* < 0.05).

To investigate the effects of PPD and PPT on the development of diabetes, we measured the body weight and food intake of high-fat diet-fed and STZ-induced diabetic mice. As shown in **Figure 3A**, body weights of mice were significantly increased when a high fat diet was fed, as compared to the NDC group from week 3 to week 4. The body weights of all STZ-injucted groups were declined gradually. After treatment period, the body weights of the HPPD and HPPT treatment groups gradually decreased as compared to the DC group. After treating for 4 weeks, HPPD treatment group showed similar features to NDC group than HPPT group.

As shown in **Figure 3B**, the food intake in high-fat diet groups was dramatically increased as compared to the NDC group during week 1 to week 4. After injecting STZ, food intake was significantly increased in PPD and PPT groups as compared with the NDC group. However, the reduction in food intake of the PPT treatment groups decreased at a slower rate than the PPD groups after 6 and 10 weeks. These data suggest that PPD and PPT can help to alleviate the diabetic symptoms arising appetite reduction.

### Effects of PPD and PPT on FBG, OGTT, and ITT

**FIGURE 4:**
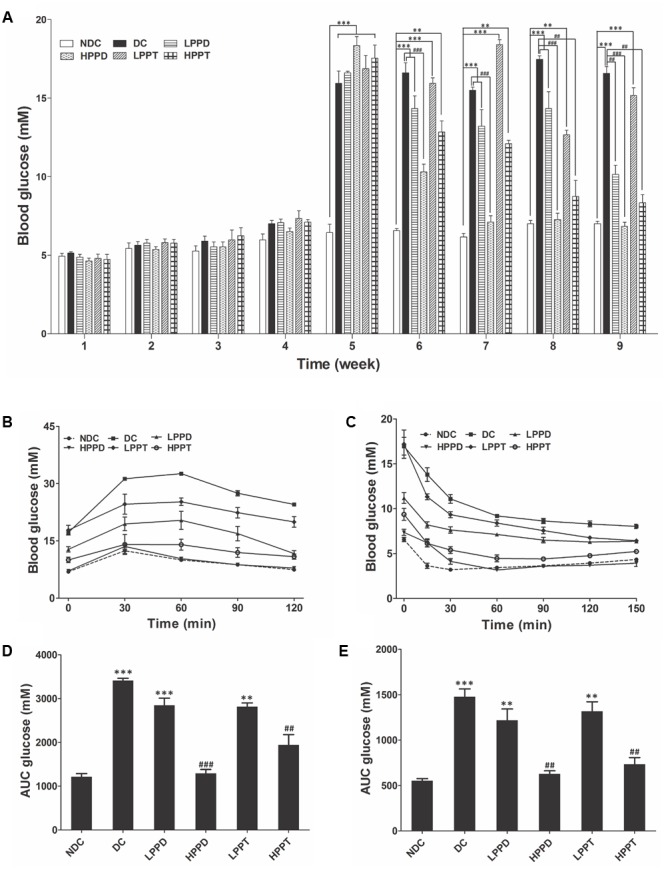
Effects of PPD and PPT on **(A)** FBG, **(B)** GTT, **(C)** ITT, and **(D,E)** AUC glucose in high-fat diet and STZ-induced diabetic mice. ^∗^*p* < 0.05, ^∗∗^*p* < 0.01, ^∗∗∗^*p* < 0.001 vs. NDC group; ^#^*p* < 0.05, ^##^*p* < 0.01, ^###^*p* < 0.001 vs. DC group. Different groups were significantly different (*p* < 0.05). ●, NDC group; ■, DC group; ▲, LPPD (PPD 50 mg/kg) group; ▼, HPPD (PPD 150 mg/kg) group; ♢, LPPT (PPT 50 mg/kg); ○, HPPT (PPT 150 mg/kg).

The FBG levels of all the groups are shown in **Figure 4A**. Initial FBG levels showed no significant difference between the NDC group and other groups during the first 4 weeks (*p* > 0.05). After injecting STZ, the FBG levels gradually increased in all DCs when compared to the NDC group (*p* < 0.001). However, PPD and PPT treatment for 4 weeks obviously reduced the FBG levels in diabetic mice. Moreover, the FBG levels in HPPD group were lower than that in HPPT group after only 2 weeks of treatment (**Figure 4A**). After 4 weeks of treatment, the FBG levels both in HPPD and HPPT groups were significantly decreased to 62.7 and 54.1%, respectively, when compared to the DC group. These data showed that PPD and PPT treatments can help to alleviate the diabetic symptom of FBG reduction.

The OGTT was performed after an overnight fast (16 h) at week 9 of the experimentation. Following oral administration of glucose, blood glucose levels reached a maximum at 30 min post-glucose gavage in the NDC group, and then gradually decreased (**Figure 4B**). While a different trend was observed in the DC group, which is indicative of glucose intolerance. Compared with the DC group, the glucose levels in PPD and PPT groups were significantly reduced in a dose dependent manner. The glucose levels in HPPD group almost reduced to similar levels in the NDC group. The corresponding AUC profile in **Figure 4B** has been shown in **Figure 4D**. The AUC values of HPPD and HPPT groups were obviously reduced 53.14 and 39.28% as compared with the DC group. These results indicated that PPD and PPT can improve glucose tolerance in diabetic mice.

To verify the effects of PPD and PPT on the ITT levels, we performed an ITT. As shown in **Figure 4C**, blood glucose levels in diabetic mice treated with PPD and PPT were significantly lower than in the DC group at 30, 60, 90, and 120 min. Moreover, the optimum dose of HPPD and HPPT groups closely resembled the curve of the NDC group. Concurrently the glucose AUC shown in **Figure 4E** revealed a marked reduction with PPD and PPT treatment when compared with the DC group (*p* < 0.001 and *p* < 0.01). As speculated, the largest reductions in glucose AUC were HPPD and HPPT treatment groups with reductions by 61.12 and 53.79%, respectively. Above results indicated that treatment with the HPPD and HPPT dramatically improved the ITT in the high-fat diet and STZ-induced diabetic mice, and that HPPD exhibited a better effect on the improvement of ITT than HPPT.

### Serum Insulin Levels, HOMA-IR, and C Peptide Levels

**FIGURE 5:**
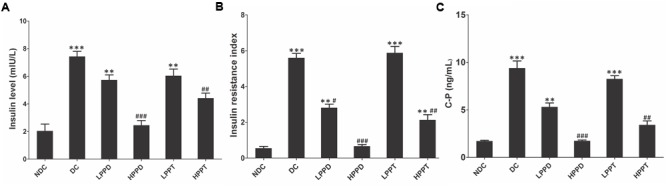
PPT and PPD ameliorates insulin level in high-fat diet and STZ-induced diabetic mice. **(A)** Serum insulin content, **(B)** HOMA-IR, **(C)** C peptide level. ^∗^*p* < 0.05, ^∗∗^*p* < 0.01, ^∗∗∗^*p* < 0.001, vs. NDC group; ^#^*p* < 0.05, ^##^*p* < 0.01, ^###^*p* < 0.001 vs. DC group. Different groups were significantly different (*p* < 0.05).

To further evaluate whether HPPD and HPPT can ameliorate diabetic phenotypes, we investigated the levels of insulin, HOMA-IR and C peptide in mice serum (**Figure 5**). Compared with the DC group, insulin and C peptide levels were significantly lower in the treatment groups given the optimum dose of HPPD (*p* < 0.001) (**Figures 5A,C**). Furthermore, a significant reduction in HOMA-IR values was observed in all PPD and PPT treatment groups when compared to the DC group, and the HPPD group showed the most robust reduction in HOMA-IR value (*p* < 0.001) (**Figure 5B**). These results indicated that PPD and PPT can ameliorate insulin resistance in diabetic mice.

### Effect of PPD and PPT on Serum Parameters

**FIGURE 6:**
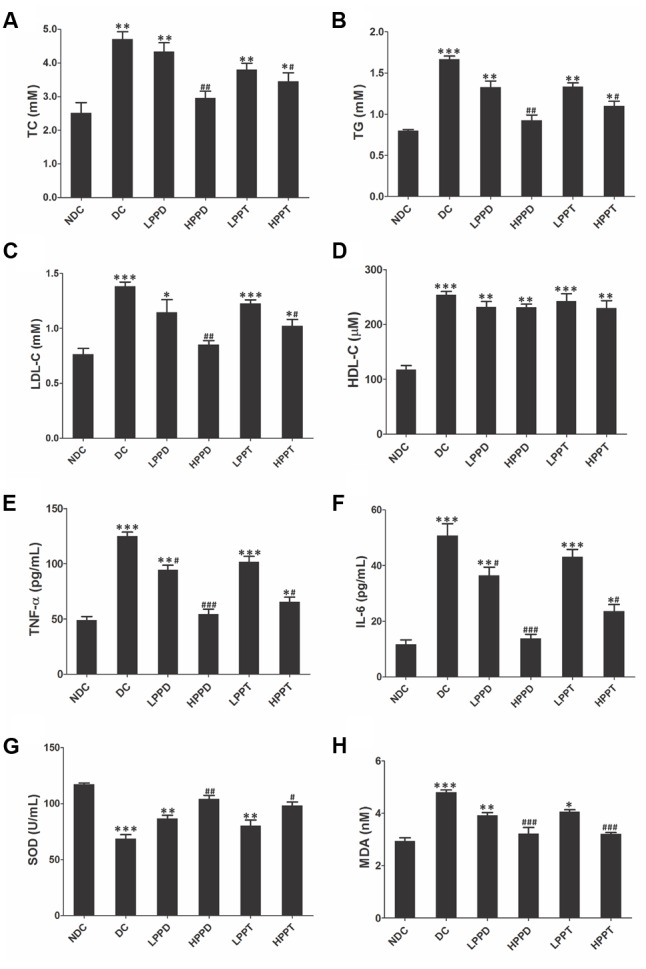
PPD and PPT ameliorates lipid disorders in high-fat diet and STZ-induced diabetic mice. **(A)** TC, **(B)** TG, **(C)** LDL-C, **(D)** HDL-C, **(E)** TNF-α, **(F)** IL-6, **(G)** SOD, **(H)** MDA. ^∗^*p* < 0.05, ^∗∗^*p* < 0.01, ^∗∗∗^*p* < 0.001 vs. NDC group, ^#^*p* < 0.05, ^##^*p* < 0.01, ^###^*p* < 0.001 vs. DC group. Different groups were significantly different (*p* < 0.05).

Next, we measured serum lipid levels to determine whether treatment with PPD and PPT can improve dyslipidemia, which is closely associated with T2DM. Serum chemistry analysis revealed that high-fat diet and STZ-induced diabetic mice exhibited significant differences in the levels of TC, TG, and LDL-C between the DC group and treatment groups. As presented in **Figures 6A–C**, treatment with PPD and PPT significantly reduced the TC, TG, and LDL-C levels as compared to the DC group (*p* < 0.01). Moreover, the TC, TG and LDL-C levels almost returned to their normal levels after treatment with a high dose of the PPD as composed with the NDC group. However, HDL-C levels were unchanged among the diabetes-induced mice groups (**Figure 6D**).

We also analyzed the secretion of inflammatory cytokines like TNF-α and IL-6 because abnormalities in inflammation are another characteristic of T2DM. Compared with the DC group, the serum levels of TNF-α and IL-6 were markedly decreased by PPD and PPT in a dose dependent manner. In the same dosage, the inhibited effects of the PPD on TNF-α and IL-6 were more than PPT (**Figures 6E,F**). These results indicated that PPD and PPT could ameliorate the inflammatory state in diabetic mice, and that PPD is better than PPT in improving inflammation.

The activities of SOD and MDA play a vital role in maintaining the balance of oxidation and anti-oxidation in the body, particularly for diabetes. Hence, we investigated the activity of SOD and MDA in the T2DM mice treated by PPD and PPT. Compared with the NDC group, the SOD activity in high-fat diet and STZ-induced mice was significantly reduced, but the activity of MDA was opposite. Treatment with PPD or PPT was observed to increase the SOD activity, and decrease the MDA activity in a dose dependent manner (**Figures 6G,H**). These results showed that PPD and PPT can improve the level of membrane lipid peroxidation in the T2DM mice.

### Histology of the Liver and Pancreas

**FIGURE 7:**
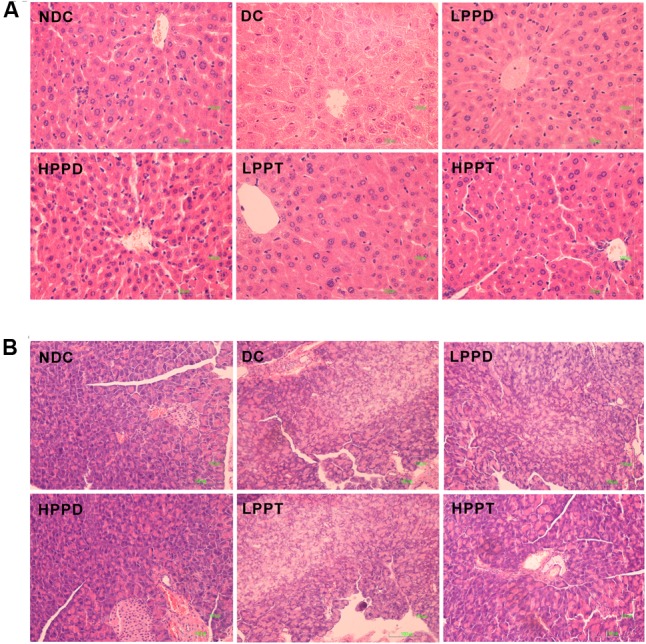
Effects of PPD and PPT on hepatic and panscrea in high-fat diet and STZ-induced diabetic mice. H&E staining of the hepatic (final magnification 400×) **(A)** and pancreas (final magnification 200×) **(B)**.

The effect of PPD and PPT on the hepatic histological changes was determined using H&E staining (**Figure 7A**). The hepatocytes showed regular distribution or nest the bands of arrangement. A typical architecture of the hepatic lobules was observed in the NDC group. In contrast, the DC group showed degenerative changes in the hepatocytes. After PPD and PPT treatment, the degeneration of the hepatocytes was significantly alleviated, and its architecture gradually returned to the normal state, especially in HPPT group. We also investigated whether there was an effect of PPD and PPT treatment on the islet cells of the pancreas. The results showed that a marked difference was observed in the number of islets cells between the DC group and the treatment groups (**Figure 7B**). Treatment with HPPD and HPPT obviously increased the number of pancreatic β-cells as compared with the DC group. We also found that the extent of cellular repair was greater with HPPD treatment as compared with HPPT treatment. Administration of HPPD and HPPT showed partial restoration of the impaired pancreatic cells due to pancreatic cell size was larger and islet cell number was increased.

### Effects of PPD and PPT on Hepatic Glucose Metabolism, Inflammation, and Lipid Metabolism

**FIGURE 8:**
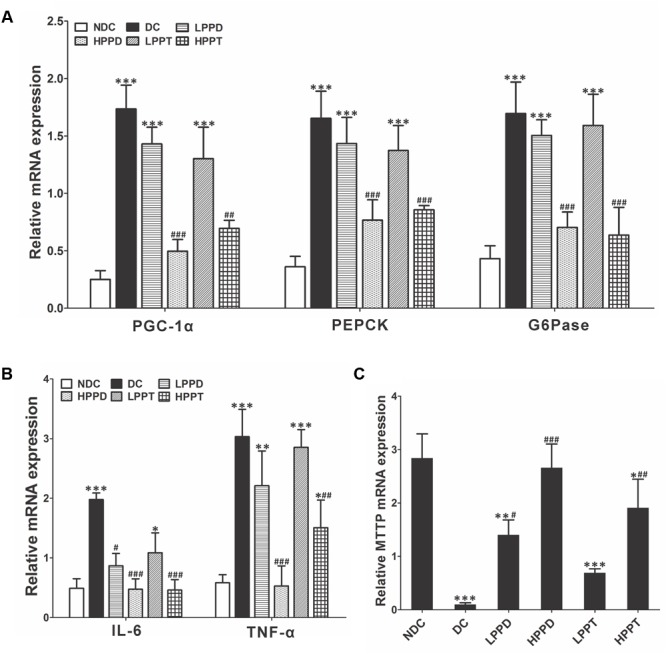
Effects of PPD and PPT in liver of relative mRNA expressions of **(A)** PGC-1α, PEPCK, G6Pase, **(B)** TNF-α, IL-6, **(C)** MTTP in high-fat and STZ-induced diabetic mice; ^∗^*p* < 0.05, ^∗∗^*p* < 0.01, ^∗∗∗^*p* < 0.001 vs. NDC group, ^#^*p* < 0.05, ^##^*p* < 0.01, ^###^*p* < 0.001 vs. DC group. Different groups were significantly different (*p* < 0.05).

To further explore the effects of PPD and PPT on diabetes, we analyzed the relative expression of key genes involved in gluconeogenesis and glycolysis. Gene expression levels of PGC-1α, PEPCK, and G6Pase were down regulated (*p* < 0.001) in the liver of mice in the HPPD and HPPT groups as compared to those in the DC group (**Figure 8A**). These results indicated that treatment with HPPD and HPPT showed a beneficial effect on abnormal hepatic glucose metabolism. We also measured the relative expression of two key markers of inflammation: TNF-α and IL-6. As shown in **Figure 8B**, a significant decrease was observed in expression of TNF-α and IL-6 in the liver of the HPPD and HPPT groups as compared with the DC group (*p* < 0.001). These data supported the notion that PPD and PPT treatment can ameliorate the T2DM-induced inflammatory responses. Furthermore, to understand whether PPD and PPT can regulate lipid metabolism, we detected the gene expression of microsomal triglyceride transfer protein (MTTP), which plays an important role in lipid transport, metabolism and secretion of lipoproteins. The relative expression of MTTP was dramatically reduced in the liver of the DC group as compared with the NDC group. However, PPD and PPT treatment significantly augmented the expression levels of MTTP in the liver of T2DM mice in a dose dependent manner (**Figure 8C**). Such results indicated that PPD and PPT may help to ameliorate the augmented T2DM-induced lipid metabolic disorder.

## Discussion

Ginsenosides, as potential active ingredients of ginseng, have been studied for their hypoglycaemic activity for many years (Hofseth and Wargovich, 2007). Some of the ginsenosides such as Rb1, Rb2, Rc, Re, Rg3, Rh2, Rg5, and compound K have been reported to possess anti-diabetic activities (Cho et al., 2006b; Lee et al., 2006; Xiong et al., 2010; Li et al., 2012c, 2016; Xiao et al., 2017). However, there is no report on comparison of anti-diabetic effects between PPD and PPT. Hence, it would be interesting to compare anti-diabetic activities of PPD and PPT. In the present study, the anti-diabetic effects of PPD and PPT were investigated using the obese-diabetic rat model by high-fat diet and low-dose STZ injection which would closely mimic the natural history of the disease events as well as the metabolic characteristics of human T2DM (Srinivasan et al., 2005).

The present work is the first to comprehensively study the effects of PPD and PPT on T2DM mice induced by high-fat diet and low-dose STZ. T2DM leads to disorders related toglucose and fatty acids metabolism. The characteristic symptoms of T2DM include an increased desire for food associated with body weight loss and hyperglycemia (Srinivasan et al., 2005; Bibak et al., 2014). In diabetic mice, the body weight decreased due to the decrease in glucose metabolism and increase in fat metabolism (Rossmeisl et al., 2003). According to our results, treatment with PPD and PPT ameliorated these parameters which were able to improve body weight and food intake at the end of the intervention period, indicating a regulatory effect on these metabolic disorders. Hyperglycemia, the most important feature of diabetes mellitus, is in itself very dangerous for diabetic patients. It impairs the prooxidant/antioxidant balance, reducing antioxidant levels and increasing free radicals (Aragno et al., 2004), which can damage the pancreatic β-cells and induce insulin resistance. Epidemiological studies (Klein et al., 1994) and clinical trials (Stolk et al., 1995) also strongly support the notion that hyperglycemia is the principal cause of complications. Our study showed that PPD exhibited significant reduction of FBG on week 2 and to reach the values of FBG showed in the NDC group. PPT also exhibited a decreasing trend after the 4 weeks treatment, although weaker than PPD. Our results demonstrated that PPD exhibit a hypoglycemic effect *in vivo*, which is in consistence with the results of previous studies with db/db mice (Lee Y.S. et al., 2010). Our results also clearly demonstrated that PPD and PPT could improve the glucose homeostasis. The OGTT levels decreased significantly after 4 weeks of treatment. More importantly, the ITT results indicated that treatment of PPD significant improved the insulin resistance. In addition, diabetes leads to a progressive accumulation of lipid metabolites. The levels of TG, TC, LDL-C, and HDL-C are considered as important biomarkers of hyperlipidemia (Bibak et al., 2014). Our results confirmed that the PPD showed a strong hypolipidemic effect as well as a hypoglycemic effect through the reduction of TG, TC, LDL-C but showed no effect on HDL-C in diabetic mice. However, these findings indicated that PPD work better than PPT. To our best knowledge, this is the first report demonstrating that PPD is better than PPT in treating T2DM. One possible reason is that PPD contains rich ginsenoside Rg5 (41.69 ± 0.92 mg/g, **Table 2**), which exhibits good anti-diabetic effect as demonstrated by our results (data not show) and other groups data (Xiao et al., 2017). In addition, oxidative stress is generally accepted as a likely causative factor in the development of insulin resistance and complications of diabetes (Giacco and Brownlee, 2010). Previous studies have shown that ginsenoside Re and fermented red ginseng extracts significantly enhance SOD activity and reduce MDA level in diabetic mice (Kim et al., 2011; Liu et al., 2012). Oxidative stress in diabetes is also associated with a reduction in antioxidant capacity, which can compound the deleterious effects of free radicals. The antioxidant enzymes such as SOD and MDA play a major role in scavenging toxic free radicals. The activity changes of these enzymes can reflect the degree of diabetic effect (Shanmugam et al., 2011). Indeed, our data showed a strong correlation between the PPD and the antioxidant activity through the improvement of the SOD and reduction of MDA activities, which are considered as markers of the antioxidant system in the organism. The high antioxidant capacity of PPD may be due to its ability of scavenging toxic free radicals.

A major cause of fasting hyperglycemia in T2DM is the increase of hepatic glucose production (Gastaldelli et al., 2006). Inhibition of hepatic glucose production contributes to glycemic control in the diabetic patients by insulin sensitizers (Lee J.M. et al., 2010). Previous studies showed that inhibition of hepatic glucose production is one of the underlying mechanisms action of metformin (Hundal et al., 2000). Our data suggest that PPD inhibits glucose production in liver. The inhibition is related to suppress the gene expressions of PGC-1α, PECPK, and G6Pase in the liver. This might be attributed to the alteration of hepatic insulin resistance (Yoon et al., 2001). Downregulation of PGC-1α, PECPK, and G6Pase in the liver improves hepatic insulin sensitivity and inhibition of hepatic glucose production in db/db mice (Koo et al., 2004). Thus, modulation of PGC-1α, PECPK, and G6Pase activities in the liver may play important roles in systemic glucose homeostasis and hepatic insulin resistance. Hepatic inflammation is an underlying cause of the pathogenesis of chronic diseases such as insulin resistance associated with T2DM (Zhang et al., 2014). Liver inflammation leads to the secretion of pro-inflammatory cytokines and chemokines, which leads to subsequent progression of these chronic diseases (Cai et al., 2005). In the present study, the degree of inflammation activation was evaluated by the expression of inflammatory mediators including TNF-α and IL-6. We found that treatment with PPD significantly down-regulated gene expressional levels of these inflammatory parameters, suggesting that the high-fat diet and STZ-induced hepatic inflammatory reactions were suppressed by PPD. To evaluate the role of the liver in the uptake and release of lipid by lipoprotein, we evaluated the gene expression of MTTP. MTTP is essential for the synthesis of VLDL in the liver. By the increased expression of MTTP, the development of steatosis can be slowed down (Wooten et al., 2016). In this study, MTTP was significantly reduced in the DC group as compared to the NDC group. Furthermore, the trends of treatment groups were same as that of the NDC group. Based on these observations, it is conceivable that the higher expression of MTTP have played a synergistic role in decreasing hepatic TG levels in HPPD and HPPT.

In most human studies, improved FBG has been known to be more important in T2DM. Ginsenoside is effective in the preventing hyperglycemia in diabetes patients (Luo and Luo, 2009). Previous study revealed that high-dose (8.68 mg/day) or long-term (4 weeks) ginsenoside treatment ameliorated diabetic conditions (Oh et al., 2014). In this study, we investigated the impact of the ginsenoside extracts, PPD and PPT, on high-fat diet and STZ-induced T2DM mice. Treatment with a relatively lower dose (3.75 mg/day) or a shorter period of time (2 weeks), can significantly improve FBG, insulin resistance, and the dyslipidemia.

## Conclusion

Our data suggest that PPD and PPT could be developed as potentially natural anti-diabetic compounds for preventing and treating the obesity-linked diabetes. After PPD and PPT treatment with the dose range between 50 and 150 mg/kg, the diabetic parameters were shown to improve in a dose-dependent manner. Unfortunately, this study was limited by a lack of sufficient mechanistic studies, a small number of subjects, and the evaluation of only short-term effects of PPD and PPT supplementation. Despite these limitations, this study is important because of the novel exploration of specific subsets of ginsenoside. Our results are promising for treating diabetes and generally consistent with other studies. Further studies with a larger number of subjects and longer duration would further broaden our knowledge regarding the same.

## Author Contributions

JD, YL, DF, and HY contributed to study design, manuscript preparation and drafting the manuscript. ZD, CZ, JH, YM, PM, and XM contributed to data collection, analysis and revising the manuscript for important content. All authors read and approved the final manuscript.

## Conflict of Interest Statement

The authors declare that the research was conducted in the absence of any commercial or financial relationships that could be construed as a potential conflict of interest.

## Abbreviations

AUC: area under the curve
DC: diabetic group
FBG: fasting blood glucose
G6Pase: glucose-6-phosphatase
H&E: hematoxylin and eosin
HPPD: 150 mg/kg bodyweight protopanaxadiol-type saponins
HPPT: 150 mg/kg bodyweight protopanaxatriol-type saponins
HDL-C: high-density lipoprotein cholesterol
HOMA-IR: insulin resistance index
HPLC: high performance liquid chromatography
IL-6: interleukin-6
ITT: insulin tolerance test
LDL-C: low-density lipoprotein cholesterol
LPPD: 50 mg/kg bodyweight protopanaxadiol-type saponins
LPPT: 50 mg/kg bodyweight protopanaxatriol-type saponins
MDA: malondialdehyde
MTTP: microsomal triglyceride transfer protein
NDC: non-diabetic group
OGTT: oral glucose tolerance
PEPCK: phosphoenolpyruvate carboxykinase
PGC-1α: peroxisome proliferator-activated receptor gamma coactivator 1-alpha
PPD: protopanaxadiol-type saponins
PPT: protopanaxatriol-type saponins
SOD: superoxide dismutase
STZ: streptozotocin
T2DM: type 2 diabetes mellitus
TG: triglyceride
TC: total cholesterol
TNF-α: tumor necrosis factor-alpha
tR: retentions times

## References

[B1] Aragno M. Mastrocola R. Catalano M. Brignardello E. Danni O. Boccuzzi G. (2004). Oxidative stress impairs skeletal muscle repair in diabetic rats. *Diabetes* 53 1082–1088. 10.2337/diabetes.53.4.1082 15047625

[B2] Basch E. Ulbricht C. Kuo G. Szapary P. Smith M. (2003). Therapeutic applications of fenugreek. *Altern. Med. Rev.* 8 20–27. 12611558

[B3] Bibak B. Khalili M. Rajaei Z. Soukhtanloo M. Hadjzadeh M. A. Hayatdavoudi P. (2014). Effects of melatonin on biochemical factors and food and water consumption in diabetic rats. *Adv. Biomed. Res.* 3 173–173. 10.4103/2277-9175.139191 25250287 PMC4166052

[B4] Cai D. Yuan M. Frantz D. F. Melendez P. A. Hansen L. Lee J. (2005). Local and systemic insulin resistance resulting from hepatic activation of IKK-β and NF-κB. *Nat. Med.* 11 183–190. 10.1038/nm1166 15685173 PMC1440292

[B5] Cho N. H. (2016). Q&A: five questions on the 2015 IDF diabetes Atlas. *Diabetes Res. Clin. Pract.* 115 157–159. 10.1016/j.diabres.2016.04.048 27242128

[B6] Cho W. C. S. Chung W.-S. Lee S. K. W. Leung A. W. N. Cheng C. H. K. Yue K. K. M. (2006a). Ginsenoside Re of *Panax ginseng* possesses significant antioxidant and antihyperlipidemic efficacies in streptozotocin-induced diabetic rats. *Eur. J. Pharmacol.* 550 173–179. 10.1016/j.ejphar.2006.08.056 17027742

[B7] Cho W. C. S. Yip T.-T. Chung W.-S. Lee S. K. W. Leung A. W. N. Cheng C. H. K. (2006b). Altered expression of serum protein in ginsenoside Re-treated diabetic rats detected by SELDI-TOF MS. *J. Ethnopharmacol.* 108 272–279. 10.1016/j.jep.2006.05.009 16797897

[B8] Gastaldelli A. Miyazaki Y. Pettiti M. Santini E. Ciociaro D. Defronzo R. A. (2006). The effect of rosiglitazone on the liver: decreased gluconeogenesis in patients with type 2 diabetes. *J. Clin. Endocrinol. Metab.* 91 806–812. 10.1210/jc.2005-1159 16352689

[B9] Giacco F. Brownlee M. (2010). “Pathogenesis of microvascular complications,” in *Textbook of Diabetes* 4th Edn eds Holt R. I. G. Cockram C. S. Flyvbjerg A. Goldstein B. J. (Oxford: Wiley-Blackwell).

[B10] Hofseth L. J. Wargovich M. J. (2007). Inflammation, cancer, and targets of ginseng. *J. Nutr.* 137 183S–185S. 17182823 10.1093/jn/137.1.183S

[B11] Hundal R. S. Krssak M. Dufour S. Laurent D. Lebon V. Chandramouli V. (2000). Mechanism by which metformin reduces glucose production in type 2 diabetes. *Diabetes Metab. Res. Rev.* 49 2063–2069. 10.2337/diabetes.49.12.2063 PMC2995498 11118008

[B12] Ji J. Zhang C. Luo X. Wang L. Zhang R. Wang Z. (2015). Effect of stay-green wheat, a novel variety of wheat in china, on glucose and lipid metabolism in high-fat diet induced type 2 diabetic rats. *Nutrients* 7 5143–5155. 10.3390/nu7075143 26132991 PMC4516991

[B13] Kim H. J. Lee S. G. Chae I. G. Kim M. J. Im N. K. Yu M. H. (2011). Antioxidant effects of fermented red ginseng extracts in streptozotocin-induced diabetic rats. *J. Ginseng Res.* 35 129–137. 10.5142/jgr.2011.35.2.129 23717054 PMC3659529

[B14] Klein R. Klein B. E. Moss S. E. Cruickshanks K. J. (1994). Relationship of hyperglycemia to the long-term incidence and progression of diabetic retinopathy. *Arch. Intern. Med.* 154 2169–2178. 10.1001/archinte.1994.00420190068008 7944837

[B15] Koo S. H. Satoh H. Herzig S. Lee C. H. Hedrick S. Kulkarni R. (2004). PGC-1 promotes insulin resistance in liver through PPAR-α-dependent induction of TRB-3. *Nat. Med.* 10 530–534. 10.1038/nm1044 15107844

[B16] Lee J. M. Seo W. Y. Song K. H. Chanda D. Kim Y. D. Kim D. K. (2010). AMPK-dependent repression of hepatic gluconeogenesis via disruption of CREB;CRTC2 complex by orphan nuclear receptor small heterodimer partner. *J. Biol. Chem.* 285 32182–32191. 10.1074/jbc.M110.134890 20688914 PMC2952219

[B17] Lee Y. S. Cha B. Y. Saito K. Yamakawa H. Choi S. S. Yamaguchi K. (2010). Nobiletin improves hyperglycemia and insulin resistance in obese diabetic ob/ob mice. *Biochem. Pharmacol.* 79 1674–1683. 10.1016/j.bcp.2010.01.034 20144590

[B18] Lee K. T. Jung T. W. Lee H. J. Kim S. G. Shin Y. S. Whang W. K. (2011). The antidiabetic effect of ginsenoside Rb2 via activation of AMPK. *Arch. Pharm. Res.* 34 1201–1208. 10.1007/s12272-011-0719-6 21811928

[B19] Lee W. K. Kao S. T. Liu I. M. Cheng J. T. (2006). Increase of insulin secretion by ginsenoside Rh2 to lower plasma glucose in Wistar rats. *Clin. Exp. Pharmacol. Physiol.* 33 27–32. 10.1111/j.1440-1681.2006.04319.x 16445695

[B20] Li K. K. Yang X. B. Yang X. W. Liu J. X. Gong X. J. (2012a). New triterpenoids from the stems and leaves of *Panax ginseng*. *Fitoterapia* 83 1030–1035. 10.1016/j.fitote.2012.05.013 22634016

[B21] Li K. K. Yao C. M. Yang X. W. (2012b). Four new dammarane-type triterpene saponins from the stems and leaves of *Panax ginseng* and their cytotoxicity on HL-60 cells. *Planta Med.* 78 189–192. 10.1055/s-0031-1280320 22034065

[B22] Li W. Yan M. Liu Y. Liu Z. Wang Z. Chen C. (2016). Ginsenoside Rg5 ameliorates cisplatin-induced nephrotoxicity in mice through inhibition of inflammation, oxidative stress, and apoptosis. *Nutrients* 8:E566 10.3390/nu8090566 PMC5037551 27649238

[B23] Li W. Zhang M. Gu J. Meng Z. J. Zhao L. C. Zheng Y. N. (2012c). Hypoglycemic effect of protopanaxadiol-type ginsenosides and compound K on type 2 diabetes mice induced by high-fat diet combining with streptozotocin via suppression of hepatic gluconeogenesis. *Fitoterapia* 83 192–198. 10.1016/j.fitote.2011.10.011 22056666

[B24] Liu Y. W. Zhu X. Li W. Lu Q. Wang J. Y. Wei Y. Q. (2012). Ginsenoside Re attenuates diabetes-associated cognitive deficits in rats. *Pharmacol. Biochem. Behav.* 101 93–98. 10.1016/j.pbb.2011.12.003 22197711

[B25] Luo J. Z. Luo L. (2009). Ginseng on hyperglycemia: effects and mechanisms. *Evid. Based Complement. Alternat. Med.* 6 423–427. 10.1093/ecam/nem178 18955300 PMC2781779

[B26] Ma S. W. Benzie I. F. Chu T. T. Fok B. S. Tomlinson B. Critchley L. A. (2008). Effect of *Panax ginseng* supplementation on biomarkers of glucose tolerance, antioxidant status and oxidative stress in type 2 diabetic subjects: results of a placebo-controlled human intervention trial. *Diabetes Obes. Metab.* 10 1125–1127. 10.1111/j.1463-1326.2008.00858.x 18355331

[B27] Niu J. Pi Z. Yue H. Yang H. Wang Y. Yu Q. (2012). Effect of 20(S)-ginsenoside Rg3 on streptozotocin-induced experimental type 2 diabetic rats: a urinary metabonomics study by rapid-resolution liquid chromatography/mass spectrometry. *Rapid Commun. Mass Spectr.* 26 2683–2689. 10.1002/rcm.6392 23124658

[B28] Oh M. R. Park S. H. Kim S. Y. Back H. I. Kim M. G. Jeon J. Y. (2014). Postprandial glucose-lowering effects of fermented red ginseng in subjects with impaired fasting glucose or type 2 diabetes: a randomized, double-blind, placebo-controlled clinical trial. *BMC Complement. Altern. Med.* 14:237 10.1186/1472-6882-14-237 PMC4227112 25015735

[B29] Park S. Ahn I. S. Kwon D. Y. Ko B. S. Jun W. K. (2008). Ginsenosides Rb1 and Rg1 suppress triglyceride accumulation in 3T3-L1 adipocytes and enhance beta-cell insulin secretion and viability in Min6 cells via PKA-dependent pathways. *Biosci. Biotechnol. Biochem.* 72 2815–2823. 10.1271/bbb.80205 18997435

[B30] Qi L. Wang C. Yuan C. (2011). Isolation and analysis of ginseng: advances and challenges. *Nat. Prod. Rep.* 28 467–495. 10.1039/c0np00057d 21258738 PMC3056508

[B31] Rossmeisl M. Rim J. S. Koza R. A. Kozak L. P. (2003). Variation in type 2 diabetes-related traits in mouse strains susceptible to diet-induced obesity. *Diabetes Metab. Res. Rev.* 52 1958–1966. 10.2337/diabetes.52.8.1958 12882911

[B32] Shanmugam K. R. Mallikarjuna K. Nishanth K. Kuo C. H. Reddy K. S. (2011). Protective effect of dietary ginger on antioxidant enzymes and oxidative damage in experimental diabetic rat tissues. *Food Chem.* 124 1436–1442. 10.1016/j.foodchem.2012.06.116

[B33] Shishtar E. Jovanovski E. Jenkins A. Vuksan V. (2014). Effects of korean white ginseng (*Panax ginseng* C.A. meyer) on vascular and glycemic health in type 2 diabetes: results of a randomized, double blind, placebo-controlled, multiple-crossover, acute dose escalation trial. *Clin. Nutr. Res.* 3 89–97. 10.7762/cnr.2014.3.2.89 25136536 PMC4135246

[B34] Sotaniemi E. A. Haapakoski E. Rautio A. (1995). Ginseng therapy in non-insulin-dependent diabetic patients. *Diabetes Care* 18 1373–1375. 10.2337/diacare.18.10.1373 8721940

[B35] Srinivasan K. Viswanad B. Asrat L. Kaul C. L. Ramarao P. (2005). Combination of high-fat diet-fed and low-dose streptozotocin-treated rat: a model for type 2 diabetes and pharmacological screening. *Pharmacol. Res.* 52 313–320. 10.1016/j.phrs.2005.05.004 15979893

[B36] Stolk R. P. Vingerling J. R. Jong P. T. V. M. D. Dielemans I. Hofman A. Lamberts S. W. (1995). Retinopathy, glucose, and insulin in an elderly population: the rotterdam study. *Diabetes Metab. Res. Rev.* 44 11–15. 10.2337/diab.44.1.11 7813804

[B37] Teng H. Chen L. Fang T. Yuan B. Lin Q. (2017). Rb2 inhibits α-glucosidase and regulates glucose metabolism by activating AMPK pathways in HepG2 cells. *J. Funct. Foods* 28 306–313. 10.1016/j.jff.2016.10.033

[B38] Wang H. Peng D. Xie J. (2009). Ginseng leaf-stem: bioactive constituents and pharmacological functions. *Chin. Med.* 4 20 10.1186/1749-8546-4-20 PMC2770043 19849852

[B39] Whiting D. R. Guariguata L. Weil C. Shaw J. (2011). IDF diabetes atlas: global estimates of the prevalence of diabetes for 2011 and 2030. *Diabetes Res. Clin. Pract.* 94 311–321. 10.1016/j.diabres.2011.10.029 22079683

[B40] Wooten J. S. Nick T. N. Seija A. Poole K. E. Stout K. B. (2016). High-fructose intake impairs the hepatic hypolipidemic effects of a high-fat fish-oil diet in C57BL/6 mice. *J. Clin. Exp. Hepatol.* 6 265–274. 10.1016/j.jceh.2016.09.001 28003715 PMC5157917

[B41] Xiao N. Yang L. L. Yang Y. L. Liu L. W. Li J. Liu B. (2017). Ginsenoside Rg5 inhibits succinate-associated lipolysis in adipose tissue and prevents muscle insulin resistance. *Front. Pharmacol.* 8:43 10.3389/fphar.2017.00043 PMC5306250 28261091

[B42] Xiong Y. Shen L. K. Tso P. Xiong Y. Wang G. Woods S. C. (2010). Antiobesity and antihyperglycemic effects of ginsenoside Rb1 in rats. *Diabetes Metab. Res. Rev.* 59 2505–2512. 10.2337/db10-0315 PMC3279544 20682695

[B43] Yoon J. C. Puigserver P. Chen G. Donovan J. Wu Z. Rhee J. (2001). Control of hepatic gluconeogenesis through the transcriptional coactivator PGC-1. *Nature* 413 131–138. 10.1038/35093050 11557972

[B44] Yuan H. D. Kim J. T. Kim S. H. Chung S. H. (2012). Ginseng and Diabetes. *J. Ginseng Res.* 36 27–39. 10.5142/jgr.2012.36.1.27 23717101 PMC3659569

[B45] Zhang X. Lv Q. Jia S. Chen Y. Sun C. Li X. (2016). Effects of flavonoid-rich Chinese bayberry (*Morella rubra* Sieb. et Zucc.) fruit extract on regulating glucose and lipid metabolism in diabetic KK-A^y^ mice. *Food Funct.* 7 3130–3140. 10.1039/c6fo00397d 27295301

[B46] Zhang Y. Yu L. Cai W. Fan S. Feng L. Ji G. (2014). Protopanaxatriol, a novel PPARγ antagonist from panax ginseng, alleviates steatosis in mice. *Sci. Rep.* 4:7375 10.1038/srep07375 PMC4260220 25487878

